# Gender differences in prevalence and associations for use of CAM in a large population study

**DOI:** 10.1186/1472-6882-14-463

**Published:** 2014-12-03

**Authors:** Agnete E Kristoffersen, Trine Stub, Anita Salamonsen, Frauke Musial, Katarina Hamberg

**Affiliations:** The National Research Center in Complementary and Alternative Medicine (NAFKAM), Department of Community Medicine, Faculty of Health Sciences, UiT The Arctic University of Norway, Tromsø, Norway; Department of Community Medicine, Faculty of Health Sciences, UiT The Arctic University of Norway, Tromsø, Norway; Department of Public Health and Clinical Medicine, Family Medicine, Umeå University, Umeå, Sweden

**Keywords:** CAM use, Norway, Gender differences, Population study, Prevalence

## Abstract

**Background:**

Self-reported use of Complementary and Alternative Medicine (CAM) varies widely from 10% to 75% in the general populations worldwide. When limited to use of a CAM provider 2% to 49% reported use is found. CAM use is believed to be closely associated with socio demographic variables such as gender, age, education, income and health complaints. However, studies have only occasionally differentiated CAM use according to gender. Therefore, the aim of the study presented here is to describe the prevalence of CAM use on the background of gender and to describe the specific characteristics of male and female users in the total Tromsø 6 population.

**Methods:**

A total of 12,982 men and women aged 30–87 in the municipality of Tromsø, Norway went through a health screening program and completed two self-administered questionnaires in 2007/2008. The questionnaires were developed specifically for the Tromsø study and included questions about life style and health issues in addition to socio demographic variables.

**Results:**

A total of 33% of the participants reported use of any CAM within the last 12 months, women more often than men (42% and 24%, respectively). When limited to visits to a CAM provider, we found 17% use among women and 8% among men. The relationship between the demographic variables and being a CAM user differed significantly between men and women with regard to age, household income, and marital status. We did not find significant differences between men and women concerning education and self-reported health.

**Conclusions:**

Findings from this study suggest that the prevalence and associations for use of CAM differ between men and women concerning several socio demographic variables (age, education and household income). Neglect of women’s health care needs in public health care may contribute to the fact that women to a higher degree than men turn to CAM and CAM products.

## Background

The use of Complementary and Alternative Medicine (CAM), including a range of different therapeutic modalities such as homeopathy, acupuncture and hands on healing, has for many years increased significantly in the Scandinavian countries [1] and elsewhere [2] but seems to have been stabilized in recent years [3, 4]. Self-reported use of any CAM in the general population varies from 10% to 75% worldwide. When restricted to visiting a CAM provider, the prevalence of use fluctuates between 2% and 49% [5, 6]. These large variations in reported use are mainly due to differences in the definition of a CAM user [7].

In 2008 16% of the Norwegian population had visited a CAM provider during the past 12 months, 22% of these were women and 9% were men [8]. In 2012 CAM was received within or outside the public health care by 36.6% of Norwegians. When use of CAM self-help techniques and CAM over the counter (OTC) products were added, 53.6% of the women and 36.5% of the men reported such use [3].

In Norway regional cohort studies have been conducted in Nord-Trøndelag (HUNT) and in Tromsø (The Tromsø Cohort study, T1-T6). In the HUNT study, visits to a CAM provider have been studied twice, in 1997 and in 2008. In 1997 a total of 9% of the respondents reported to have visited a CAM provider during the last 12 months. The proportion was 13% in 2008 [9]. In the Tromsø study, the use of CAM was investigated in the 3^rd^ (T3), 4^th^ (T4), 5^th^ (T5), and 6^th^ (T6) study conducted in 1986/87, 1994/95, 2001/02 and 2007/08, respectively. The use of a CAM provider during the last 12 months in the 5^th^ Tromsø study was found to be similar to the results in HUNT (1997) [10].

Based on data from large cross sectional surveys, the use of CAM is believed to be closely associated with socio demographic variables such as gender, age, education, income and health complaints [5]. Issues such as control and participation in treatment, perception of illness, natural treatment, holism and philosophy of life have been found to be related to the use of CAM [11, 12].

Over the last decades researchers in the medical field have shown a growing interest in gender and differences between men and women in health, disease and health behaviour [13, 14]. Research has shown that factors such as different biological processes, conditions in daily life, environmental experiences, risk behaviours and different responses to stressful events may contribute to variation in health and well-being in men and women [13–15]. There is also evidence that women are seldom offered the same treatment as men, even though they suffer the same medical conditions, and there was no medical or clinical reason for the treatment choice. Moreover, studies show that women are less likely than men to receive more advanced diagnostic and therapeutic interventions [16–18]. This phenomenon raises the question of gender bias.

In the field of CAM research there are few studies focusing on gender issues, but studies generally demonstrate that men use CAM to a lesser degree than women. Studies focusing on CAM use often focus on particular patient populations, for example patients with pain or different types of cancer. As a matter of fact, measuring gender differences in CAM use based on a few disorders may be associated with a gender bias because the disorders may have different prevalence with regard to gender. Only large prospective population studies, such as the Tromsø study, have the potential to reveal an unbiased perspective with regard to gender differences of CAM use in the total population. However, this topic has rarely been studied in great population studies. Therefore, the aim of the study presented here is to describe the prevalence and characteristics of male and female users of CAM in the total Tromsø 6 population.

## Methods

A total of 12,982 men and women aged 30–87 in the municipality of Tromsø, Norway went through a health screening program and completed two self-administered questionnaires in 2007/2008.

The Tromsø Study series (1–6) are single-centered prospective, population-based health surveys of the adult inhabitants of the municipality of Tromsø, North Norway [19]. The population of Tromsø reflects the distribution of gender, educational level and average income in Norway overall, but the population is somewhat younger [20]. The design includes repeated population health surveys to which total birth cohorts and random samples are invited. The letter of invitation contained a short questionnaire. Individuals, who attended the survey by undergoing a health screening and answered the first questionnaire, subsequently received a second, more detailed questionnaire that they were asked to complete and return at site or later by mail.

The two questionnaires included questions on the general health status, diseases suffered by the respondent or their family, muscle pain and physical discomfort, food habits, alcohol consumption, smoking habits, physical activity in leisure time, level of education, use of medicine and use of health services including CAM. The questions regarding CAM use were not related to any specific disease condition. The questionnaires have not been validated, however, a Cronbach’s alpha test of internal consistency of the questions analyzed in this paper was 0.403.

The health screening included measuring of height, weight, waist and hip circumference and blood pressure. A blood sample was also taken to measure serum total cholesterol, HDL-cholesterol, triglycerides and glucose.

In Norway a CAM provider is understood as a practitioner providing CAM both as alternative and complementary treatment. A CAM provider offers therapies that are mostly offered outside the public health care service and paid out-of-pocket by the patients themselves when fees are required.

Study participants were classified as “CAM-users” by checking YES for one or more of the following questions:

1. *Have you during the last 12 months seen an alternative provider* (*homeopath*, *acupuncturist*, *foot zone therapist*, *herbal medicine practitioner*, *laying on of hands practitioner*, *healer*, *clairvoyant etc*.)?
2. *In the last 12 months*, *have you used herbal or* “*natural*” *medicine*?
3. *In the last 12 months*, *have you used meditation*, *yoga*, *qi gong or Tai Chi as self*-*treatment*?

Accordingly, a participant who checked NO for all the three specific CAM-questions was classified as a non-user. The parenthesis examples in question 1 include examples of both modern CAM modalities and traditional healing as it is practiced in North Norway. Traditional healers are non-professional and non-commercial therapists who are closely connected to the local culture and world-views [21, 22].

CAM use is reported at levels 1–3 in the The National Research Center in Complementary and Alternative Medicine (NAFKAM) 6 level cumulative model for classifying CAM use [7]. In this model the different levels are included in each other (Table 1).

**Table 1:** **Gender differences in CAM use last 12 months according to NAFKAMS cumulative model for reporting CAM use**

	Total (n = 11104) %	Women (n = 5877) %	Men (n = 5227) %	p-value*
**CAM 1** (seen a CAM provider more than 3 times)	(n = 535) 4.8%	(n = 353) 6%	(n = 182) 3.5%	<0.000
**CAM 2** (seen a CAM provider at least once)	(n = 1423) 13.1%	(n = 995) 17.4%	(n = 428) 8.3%	<0.000
**CAM 3** (seen a CAM provider, used OTC products or CAM techniques)	(n = 3730) 33.3%	(n = 2471) 42.0%	(n = 1259) 24.1%	<0.000

*Pearson Chi-Square test.

Informants who had seen a chiropractor were not defined as CAM users in this study as chiropractors are regulated health care personnel in Norway. This also applies to informants who had used cod liver oil, Omega-3 capsules, calcium tablets or ordinary vitamins/mineral supplements as these supplements are commonly used in the Norwegian population.

The data were analyzed using chi-square tests and Anova Table in SPSS Windows (version 19.0, SPSS Inc., Chicago, IL).

The Norwegian Data Protection Authority has been notified about the study, and The Regional Committee for Medical and Health Research Ethics has recommended it. The participants have given their informed, written consent.

## Results

### Basic characteristics of the studied participants

**Figure 1:**
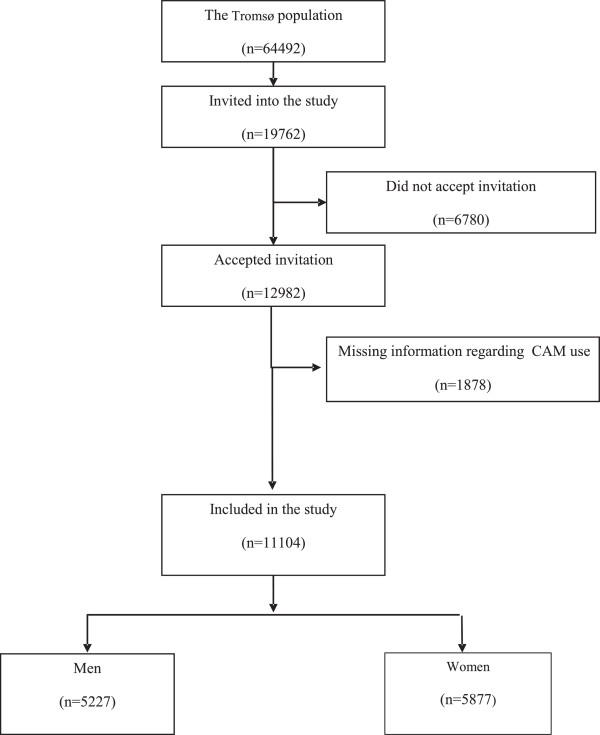
**Flow chart that shows the selection of the studied population.**

The studied population (n = 11104) consisted of 5,877 women (52.9%) and 5,227 men (47.1%). The response rate to the study was 65.7%. After exclusion of missing response to the CAM questions, the response rate reached 56%, 58% among women and 54.3% among men Figure 1.

The studied population was a rather healthy and gender balanced population with a median age of 58. More than 92% defined themselves as being of Norwegian heritage. The largest majority of the remaining 8% defined themselves as being of indigenous Sami people and/or Kven, descendants of Finnish immigrants. The largest differences between men and women were with regard to income, partner and visits to a general practitioner (GP) (Table 2).

**Table 2:** **Basic characteristics of the studied participants**

	Total (n = 11104)	Women (n = 8577)	Men (n = 5227)	p-value
Percentage women	52.9			
Mean age	56.75	56.58	56.94	0.118^2^
Median age (range)	58 (30–87)	58 (30–87)	59 (30–87)	
Living with a spouse/partner %	75.9	69.8	82.5	<0.000^1^
University degree %	39.9	38.8	41	<0.000^1^
Self-reported good health %	67	66.2	68	<0.000^1^
Self-reported poor health %	5.2	5.8	4.5	<0.000^1^
Mean self-reported health (0–100)	77.5	77.1	78	0.032^2^
Median self-reported health (0–100)	80	80	80	
More than 400 000 NOK in household income during past 12 months % (69 000$/52000€)	62.9	56.9	69.4	<0.000^1^
Less than 125 000 NOK (21 500 $/16 400 €) in household income during past 12 months %.	2.5	3.6	1.3	<0.000^1^
Seen a GP during past 12 months %	82.2	86	77.9	<0.000^1^

^1^Pearson Chi-Square test.

^2^Anova test.

### Gender differences in prevalence of CAM use

A total of 1,423 participants (13.1%) had seen a CAM provider at least once within the past year (CAM level 2) of which 535 participants (4.8%) had seen the provider more than three times (CAM level 1). A total of 3,730 (33.3%) had seen a CAM provider, used CAM OTC-products and/or used CAM techniques (CAM level 3) within the past year. Women used CAM significantly more often than men at all levels (Table 1).

### Gender related associations for CAM use

The relationship between the demographic variables and being a CAM user differed significantly between men and women with regard to age (CAM level 3), household income (CAM levels 2 and 3) and marital status (CAM levels 1–3). We did not find significant differences between men and women concerning education or self-reported health (Table 3).

**Table 3:** **Socio demographic characteristics with comparison between female and male users of CAM**

	CAM 1 ^1^			CAM 2 ^2^			CAM 3 ^3^		
	Women	Men		Women	Men		Women	Men	
	% (n)	% (n)	p-value*	% (n)	% (n)	p-value*	% (n)	% (n)	p-value*
**Age**			0.195			0.318			**0.000**
30-39	7.4 (26)	4.9 (9)		5.7 (57)	4.4 (19)		4.2 (105)	2.9 (37)	
40-59	58.6 (207)	53.8 (98)		53.9 (536)	51.4 (220)		52.5 (1297)	44.1 (555)	
60+	34 (120)	41.2 (75)		40.4 (402)	44.2 (189)		43.3 (1069)	53 (667)	
**Education**			0.441			0.569			0.268
Primary/Secondary	52.3 (183)	55.8 (101)		59.9 (591)	61.6 (261)		58.5 (1432)	60.4 (751)	
College/University education	47.7 (167)	44.2 (80)		40.1 (395)	38.4 (163)		41.5 (1017)	39.6 (493)	
**Self**-**reported health**			0.984			0.962			0.439
Bad	8.2 (29)	8.2 (15)		10.3 (102)	10.4 (44)		7.9 (194)	6.8 (85)	
Neither good nor bad	30.6 (108)	31.3 (57)		33.8 (333)	33 (140)		31 (759)	32.1 (400)	
Good	61.2 (216)	60.4 (110)		55.9 (551)	56.6 (240)		61.1 (1499)	61.1 (763)	
**Marital status**			**0.000**			**0.000**			**0.000**
Single	21.2 (75)	24.2 (44)		18.8 (187)	24.1 (103)		17.3 (427)	18.7 (236)	
Married/Registrated partner	52.1 (184)	64.3 (117)		51.9 (516)	62.6 (268)		54.9 (1356)	64.3 (810)	
Widow/Widower	9.3 (33)	1.6 (3)		11.1 (110)	2.1 (9)		10.3 (254)	4.2 (53)	
Divorced/Separated	17.3 (61)	9.9 (18)		18.3 (182)	11.2 (48)		17.6 (343)	12.7 (160)	
**Household income**	.		0.331			**0.005**			**0.012**
Low	24.5 (80)	18.5 (33)		26.6 (243)	18.4 (75)		26 (590)	21.5 (261)	
Middel	33 (108)	36 (64)		34 (310)	36.4 (148)		33.8 (766)	34.9 (423)	
High	42.5 (139)	45.5 (81)		39.4 (360)	45.2 (184)		40.2 (913)	43.6 (528)	

^1^CAM 1 = Use of a CAM provider minimum 4 times last year.

^2^CAM 2 = Use of a CAM provider last year.

^3^CAM 3 = Use of a CAM provider, CAM OTC products or CAM techniques last year.

*Pearson Chi-Square test.

Comparison between female and male users of CAM.

In the sex-disaggregated analysis, we found that poor health, young or middle age was significantly associated with CAM use at all CAM levels in women. We also found university education to be associated with CAM use at levels 1 and 3, and that divorced/separated women tended to use more CAM at level 3. We did not find household income to be associated with CAM use at any level among women (Table 4).

**Table 4:** **Socio demographic characteristics of female users and nonusers of CAM**

	CAM 1 ^1^			CAM 2 ^2^			CAM3 ^3^		
	Non-users	Users		Non-users	Users		Non-users	Users	
	% (n)	% (n)	p-value*	% (n)	% (n)	p-value*	% (n)	% (n)	p-value*
**Age**			**0.000**			**0.000**			**0.000**
30-39	90 (234)	10 (26)		78.2 (205)	21.8 (57)		60.7 (162)	39.3 (105)	
40-59	92.4 (2534)	7.6 (207)		80.8 (2262)	19.2 (536)		54.9 (1576)	45.1 (1297)	
60+	95.3 (2423)	4.7 (120)		84.8 (2248)	15.2 (402)		60.9 (1668)	39.1 (1069)	
**Education**			**0.001**			0.434			**0.000**
Primary/Secondary	94.5 (3140)	5.5 (183)		82.9 (2858)	17.1 (591)		59.7 (2124)	40.3 (1432)	
College/University education	92.3 (1999)	7.7 (167)		82.1 (1806)	17.9 (395)		54.9 (1238)	45.1 (1017)	
**Self**-**reported health**			**0.012**			**0.000**			**0.000**
Bad	90.2 (266)	9.8 (29)		67.8 (215)	32.2 (102)		42.8 (145)	57.2 (194)	
Neither good nor bad	92.9 (1413)	7.1 (108)		78.9 (1244)	21.1 (333)		53.4 (870)	46.6 (759)	
Good	94.2 (3478)	5.8 (216)		85.4 (3226)	14.6 (551)		61.2 (2367)	38.8 (1499)	
**Marital status**			0.172			0.084			**0.006**
Single	92.3 (902)	7.7 (75)		81.2 (808)	18.8 (187)		57.9 (588)	42.1 (427)	
Married/Registrated partner	94 (2877)	6 (184)		83.5 (2613)	16.5 (516)		57.9 (1866)	42.1 (1356)	
Widow	94.7 (587)	5.3 (33)		83.3 (550)	16.7 (110)		63.1 (435)	36.9 (254)	
Divorced/Separated	93.1 (825)	6.9 (61)		80.3 (744)	19.7 (182)		54.4 (517)	45.6 (434)	
**Household**			0.711			0.510			0.101
Low	94 (1264)	6 (80)		82.7 (1161)	17.3 (243)		59.3 (861)	40.7 (590)	
Middel	93.5 (1541)	6.5 (108)		81.7 (1383)	18.3 (310)		55.7 (963)	44.3 (766)	
High	93.6 (1959)	6.6 (139)		83.1 (1771)	16.9 (360)		58.1 (1266)	41.9 (913)	

^1^CAM 1 = Use of a CAM provider minimum 4 times last year.

^2^CAM 2 = Use of a CAM provider last year.

^3^CAM 3 = Use of a CAM provider, CAM OTC products or CAM.

Techniques last year.

*Pearson Chi-Square test.

Among men we found that poor health was associated with CAM use at all CAM levels while older age, lower income and being a widower was associated with CAM use at CAM level 3 (Table 5).

**Table 5:** **Socio demographic characteristics of male users and nonusers of CAM**

	CAM 1 ^1^			CAM2 ^2^			CAM3 ^3^		
	Non-users	Users		Non-users	Users		Non-users	Users	
	% (n)	% (n)	p-value*	% (n)	% (n)	p-value*	% (n)	% (n)	p-value*
**Age**			0.154			0.202			0.001
30-39	95.3 (181)	4.7 (9)		90 (171)	10 (19)		80.5 (153)	19.5 (37)	
40-59	96 (2365)	4 (98)		84.8 (2272)	8.8 (220)		77.9 (1953)	22.1 (555)	
60+	96.9 (2370)	3.1 (75)		92.4 (2295)	7.6 (189)		73.6 (1862)	26.4 (667)	
**Education**			0.416			0.255			0.245
Primary/Secondary	96.6 (2864)	3.4 (101)		91.3 (2755)	8.7 (261)		75.4 (2300)	24.6 (751)	
College/University education	96.2 (2004)	3.8 (80)		92.2 (1937)	7.8 (163)		76.8 (1631)	23.2 (493)	
**Self**-**reported health**			**0.008**			**0.000**			**0.000**
Bad	93.2 (204)	6.8 (15)		80.7 (184)	19.3 (44)		63.4 (147)	36.6 (85)	
Neither good nor bad	95.9 (1322)	4.1 (57)		90 (1267)	10 (140)		72.1 (1032)	27.9 (400)	
Good	96.8 (3361)	3.2 (110)		93.1 (3262)	6.9 (240)		78.4 (2769)	21.6 (763)	
**Marital status**			0.240			0.074			**0.023**
Single	95.6 (950)	4.4 (44)		89.8 (907)	10.2 (103)		76.7 (779)	23.3 (236)	
Married/Registrated partner	96.5 (3199)	3.5 (117)		92 (3088)	8 (268)		76.2 (2587)	23.8 (810)	
Widower	97.6 (143)	2.1 (3)		93.9 (139)	6.1 (9)		65.6 (101)	34.4 (53)	
Divorced/Separated	97.2 (624)	2.8 (18)		92.6 (604)	7.4 (48)		75.8 (501)	24.2 (160)	
**Household income**			0.484			0.135			**0.000**
Low’	96.2 (835)	3.8 (33)		91.5 (809)	8.5 (75)		71.1 (642)	28.9 (261)	
Middel	96 (1539)	4 (64)		90.9 (1472)	9.1 (148)		74.2 (1219)	25.8 (423)	
High	96.7 (2375)	3.3 (81)		92.6 (2297)	7.4 (184)		78.9 (1969)	21.1 (528)	

^1^CAM 1 = Use of a CAM provider minimum 4 times last year.

^2^CAM 2 = Use of a CAM provider last year.

^3^CAM 3 = Use of a CAM provider, CAM OTC products or CAM technuiques last year.

*Pearson Chi-Square test.

Common in both men and women was that poor health was associated with CAM use at all levels. At levels 1 and 2, the CAM users tended to be younger compared to the non-user in both men and women, while the oldest group was the most frequent CAM user among men at level 3 (Tables 4 and 5).

While lower income is associated with CAM use at level 3 for men, income was not associated with CAM use at all among women (Tables 4 and 5).

## Discussion

Results from this study indicate that women were generally more likely to use CAM at all levels. For both men and women, poor health was associated with CAM utilization at all levels. Moreover, younger age was associated with the use of CAM at all levels for women. Household income in women and university education in men were not associated with the use of CAM at any level.

Many studies report use of CAM in populations. However, the study population, time frame in use and definition of CAM vary [7]. Emphasizing comparability between studies, we have chosen to compare our study to a limited selection of other studies.

The most comparable study is the HUNT 3 study conducted in the county of Nord-Trøndelag, Central Norway in 2008. They found similar use at CAM level 2 as shown in the results from our study [9].

The previous Tromsø study (the 5^th^ Tromsø study), conducted in 2001/2002 reported lower use of CAM at level 2 compared to results from our group six years later (13.5% in women and 4.8% in men without cancer or coronary heart disease) [10]. The higher use of CAM in this study might be due to a general increase in the use of CAM between 2002 and 2008 [6, 23]. The pre-prepared list regarding the CAM providers might have improved the recall and clarified what to consider as CAM, and thereby increased the reported rate of CAM use [6].

The findings of 33.3% use at CAM level 3 are in accordance with a recent review that found 32.2% prevalence of use in the 16 studies included [2]. The findings from our study are also in accordance with findings from the UK and the US presented in another recent review [5]. However, our data report a lower prevalence of use than findings from Australia and South Korea [5]. The reason for this might be that South Korea has a stronger tradition of using traditional medicine, and that Australia is more influenced by traditional Eastern medicine due to geographical proximity.

The findings of a strong association between CAM use and being a young/middle age women with higher education and poor health, is in line with other studies [1, 24–26]. That women in this study used CAM more often than men at all levels was also in line with previous research, showing that women tend to be more active in their own health promotion and are more concerned about health issues and caring compared to men [13, 27]. Throughout the Western world women more often undertake health care visits [28–30], are more frequent utilizers of psychological support groups [31, 32] and of health advice on the Internet [33], and the typical reader of a self-help book is a woman. The presence of a gender bias in health care, which can lead to a neglect of women’s health care needs [17], might also contribute to women turning away from conventional patterns of care towards CAM providers and products.

Recent in-depth studies among Norwegian women using CAM support the findings that the public healthcare system in Norway may be unable to meet all the needs of female patients. The results illustrate subjectively experienced barriers related to the communication, understanding, and treatment of illness. Women diagnosed with breast cancer and multiple sclerosis express unmet needs with regard to their individual health care goals. They strongly emphasize the importance of CAM as a health care system that enables them to take active part in decision-making processes and treatment and, thereby, contribute to positive health outcomes for themselves. They not only relate to scientific, medical knowledge but also to experience-based knowledge (e.g., bodily experiences) [34, 35] as an important basis for their treatment decisions. By the decision to use CAM, and even in some cases to delay or decline conventional treatment, female CAM users differ from the expected patient behavior and challenge the rationality of medical advice which traditionally has been defined and provided by men [34, 35].

However, another reason for the higher use in women might be the fact that women generally utilize more health services than men [13, 36]. Together with the potential implications gender bias in healthcare has for women [17], this would explain why generally more women feel the need to seek alternatives to conventional care.

Women’s widespread use of CAM in Western countries has also been suggested as an expression of traditional gender roles and dominant discourses of femininity, as being “help-seeking” and adhering to the “patient role” is more in coherence with a traditional femininity than masculinity [37, 38]. The lower use of CAM among men may be explained by the fact that men adapt to preconceptions about a masculine behavior with little room for showing weakness and a need for help and support [27], and potentially the fact that their health care needs are better met within the public health care [17]. Another explanation is related to ideas about men perceiving their body and health as being more “mechanical” than women, and that they, therefore, are less attracted to CAM where wholeness, communication and personal relation are more pronounced than the detailed biological mechanism [38].

The higher education among women using CAM was expected to correlate with the younger age. Analyzes adjusted for age, however, still show significant associations related to education. Women with higher education might be more able to find relevant information about CAM and to afford such treatment use. It can be speculated, that the higher educational background of women with a university education leads to higher self-confidence especially since even in modern Norway, it is not entirely the rule that women and men have the same education, in particular not for all of the age groups in this sample. It is not unlikely that this higher self-esteem makes females less apt to accept terms and conditions of treatment that they do not feel entirely comfortable with.

The lack of income differences between the users and non-users of CAM is in line with several other studies [26]. Recent studies suggest that CAM no longer is a phenomenon restricted to unique segments enjoying high family income [39].

### Limitations

Despite the large sample size, the response rate (65.7%) could have influenced the generalizability of our findings as the non-responders differed from the responders concerning age and sex. Men in the oldest and youngest age groups and women in the oldest age group had the lowest response rate. However, the non-responders in these groups consist of only 391 responders. Therefore, even though these groups would have been expected to use CAM to a lesser degree, the number of non-responders in these groups is so low that the likelihood of substantially influencing the final results is small.

The generalizability was also influenced by the 1,878 respondents that were excluded from the study due to missing response to the CAM questions. Generally, non-response to a CAM question is more likely to mean no use leading to an overestimated use of CAM in the analysis [40]. A non-responder-analysis, however, where the non-responders to the CAM questions are assumed to not having used CAM, showed that the tendencies are the same, though the significant level varies slightly.

The 12-month recall period concerning the use of CAM, might result in inaccuracies regarding the report of use. However, this factor is equally distributed between men and women.

One of the three questions regarding CAM asked for the use of herbal or “natural medicine” without defining this further. This could constitute an over- or underreporting of such use depending on how each participant defined their use. Moreover, young and old participants, men and women, might define this in different ways.

Traditional healing and CAM therapies might have different prevalence and associations for use. Both are combined in the same question, which makes a differentiation between associations for CAM and traditional healing, difficult.

## Conclusion

Findings from this study suggest that the prevalence and associations for use of CAM differ between men and women concerning several socio demographic variables (age, education and household income). Norwegian women use CAM significantly more often than men at all levels of the use of CAM. The study revealed a strong association between the use of CAM and being a young/middle aged women with higher education and poor health.

## Abbreviations

CAM: Complementary and Alternative Medicine
NAFKAM: National Research Center in Complementary and Alternative Medicine
OTC: Over the counter.
